# Shadow-Imaging-Based Triangulation Approach for Tool Deflection Measurement

**DOI:** 10.3390/s23208593

**Published:** 2023-10-20

**Authors:** Marina Terlau, Axel von Freyberg, Dirk Stöbener, Andreas Fischer

**Affiliations:** 1 University of Bremen, Bremen Institute for Metrology, Automation and Quality Science, Linzer Straße 13, D-28359 Bremen, Germanyd.stoebener@bimaq.de (D.S.); andreas.fischer@bimaq.de (A.F.); 2 MAPEX Center for Materials and Processes, University of Bremen, D-28334 Bremen, Germany

**Keywords:** incremental sheet forming, triangulation, shadow imaging, three-dimensional position measurement

## Abstract

As incrementally formed sheets show large geometric deviations resulting from the deflection of the forming tool, an in-process measurement of the tool tip position is required. In order to cover a measuring volume of 2.0 m × 1.0 m × 0.2 m and to achieve measuring uncertainties of less than 50 µm, a multi-sensor system based on triangulation is realized. Each shadow imaging sensor in the multi-sensor system evaluates the direction vector to an LED attached to the tool, and the three-dimensional position of the LED is then determined from the combination of two sensors. Experimental results show that the angle of view from the sensor to the LED limits both the measurement range and the measurement uncertainty. The measurement uncertainty is dominated by systematic deviations, but these can be compensated, so that the measurement uncertainty required for measuring the tool tip position in the ISF is achieved.

## 1. Introduction

### 1.1. Motivation

In comparison with conventional forming processes, incremental sheet forming (ISF) is an economical alternative for forming large sheet metals in small lot sizes [1]. Since a universally applicable forming stylus forms the sheet metal over a counter die with an arbitrary shape [2], the machine tool costs are significantly reduced. However, one disadvantage of ISF is that major geometrical deviations occur due to spring back [3] and tool deflection [4]. To enable the compensation of the tool deflection, it must be determined. For this purpose, a prediction of the tool deflection using mechanical calculations is performed [5]. However, these predictions are based on model assumptions and do not consider the machine tool error or deformations of the machine tool. Therefore, a tool deflection measurement is preferred instead.

The required tool deflection measurement system must be capable of measuring the three-dimensional tool tip position in the ISF process. So, the measurement system has to work contactless and fast, capturing the tool position close to the tool tip in a single shot. Additionally, the measurement system should be independent of the machine tool kinematics.

### 1.2. State of the Art

To meet the requirements for an in-process deflection measurement of the moving tool, optical measurement systems are reasonable approaches. In the intended application, a machining volume of 2.0 m × 1.0 m × 0.2 m is covered by the measurement system. To enable the detection of typically occurring tool deflection of 150–450 µm, a measurement uncertainty of ≤50 µm is targeted. Consequently, a challenging dynamic range (i.e., measurement range divided by measurement uncertainty) of $4\times 10^{4}$ is required. Covering the entire measuring volume with a global measurement approach, e.g., full-field photogrammetry, the dynamic range is not achievable [6]. On the contrary, a local measurement approach, e.g., tracking laser interferometry, is capable of achieving the required dynamic range. Therefore, laser trackers are usually applied to measure the machine tool error [7]. Even in robotic ISF, a laser tracker is applied to measure the tool center position in order to control the forming process in real time [8]. However, to determine the tool deflection and the machine deformation, a reflector must be attached close to the tool tip. The tracked reflector at the tool tip might move out of the system’s field of view during a loss of view and then the tracking system fails. As a compromise between the local and the global measurement approach without scanning, a multi-sensor approach is proposed, where several sensors are arranged around the measuring volume and each sensor covers a small sub-region of the full volume [9]. Applying the multi-sensor approach, a setup that is robust to a loss of view is realizable and an enhanced dynamic range is achievable.

Since the sensors must be located outside the machining volume, an applicable sensor technology has to cover a sub-region of an axial measuring range of 500 mm, which is half the width of the machining volume. Additionally, a lateral measuring range in the horizontal and vertical direction of 200 mm is aimed for. The sensor implementation at the machine tool could be realized, e.g., on the edge of the worktable or on a separate frame around the machine tool whereby the sensors might need to be oriented at an certain angle so that the machine tool or the clamping does not cover the machining area. Considering the time resolution, a measurement duration below 1 ms is required so that the LED moves only 50 µm during the measurement and motion blur is kept sufficiently low when operating at a common feed rate of 50 mm/s [10]. For this purpose, the position has to be captured in a single shot. To provide a new position measurement at each 1 mm tool movement, a measuring rate of 50 Hz is necessary. Resulting from these requirements, camera-based methods determining a position via triangulation with an angle-of-view measurement are suitable, because they provide an appropriate field of view, and exposure times below 1 ms and frame rates above 50 Hz are feasible.

Photogrammetry is a particularly suitable approach for measuring the 3D positions of multiple points with reflector targets [11]. Industrial applications include deformation measurements, i.e., displacement field measurements of the object’s surface, of a model in a wind tunnel [12] or of a wind turbine blade in static and fatigue tests [13]; alignment of row parts before machining [14]; or the tracking of robot end-effectors [15]. Although tracking robot end-effectors is a similar application to tool tip measurement, transferring the measurement principle is neither practical, due to the use of reflector targets that are too large to be placed close to the tool tip, nor does it reach a sufficient dynamic range [6]. For photogrammetric shape measurement, artificial patterns are pasted on the surface of the measuring object [11]. Here, the 3D shape is obtained using stereoscopic digital image correlation (DIC). Three-dimensional DIC was applied, for example, for the analysis of three-dimensional displacement fields in fracture experiments [16] or in ISF for measuring the shape of the formed part to iteratively control the forming process [17]. Siebert et al. [18] have shown that 3D DIC enables a sufficient dynamic range in the lateral but not in the axial direction with respect to the intended application in tool deflection measurement. Another approach to measure 3D displacement fields using only a single camera is based on laser speckles. Using speckle photography, Tausendfreund et al. [19] measured 3D displacement fields during the deep rolling process. To achieve a high spatial resolution, the field of view of the camera is less than 10 mm wide, which is a too-small measurement range to cover a sufficient part of the machining volume in ISF. Therefore, due to the larger field of view, photogrammetric approaches seem more suitable for measuring the tool tip position in ISF. However, photogrammetric measurement is based on tracking features, which can be, e.g., a surface texture, the edges of an object or markers attached to or painted on the object surface, i.e., the tool tip surface. As a result, the information is only contained in a few of more than a million pixels in the image.

In order to maximize the image information content and to use the full image frame of the camera, Grenet et al. introduced a shadow imaging concept to measure the position of a light source [20]. In shadow imaging, the light source casts a shadow through a mask in front of a camera chip and the light source position is calculated from the image of the shadow. Thereby, the lateral position is obtained from the shadow position and the axial position can either be calculated from the magnification of the shadow pattern or from triangulating the shadow positions of at least two sensors. Although the shadow of a moving light source is recorded in in-process measurement, the shadow position shift, i.e., the motion blur, during a single shot measurement can be kept sufficiently low, at less than 1 pixel, by using an appropriate sensor design. To enable an absolute three-dimensional light source position measurement, i.e., an absolute two-dimensional shadow position evaluation, a checkerboard pattern with absolute coding or a center feature is proposed. Another pattern with absolute and two-dimensional features was created by André et al. [21], which contains periodic arranged squares and binary absolute coding. The pattern is applied as a micro-encoded target and the target’s in-plane position is measured.

In summary, it stands out that stereo- and multi-camera systems, which are robust to failure, have not been used for measuring the tool tip position in ISF yet. Since the measurement uncertainty decreases with an increasing feature content in the image [22], the shadow imaging principle is pursued here for application in ISF. Previous work has shown that shadow imaging is capable of achieving the required tool tip position measurement uncertainty of the lateral position components, but also that the required dynamic range of the axial position component is not achievable using a single shadow imaging sensor [23]. It was shown that the random error of the axial position measurement, which deteriorates as the measurement distance increases, exceeds 250 µm in a distance of 500 mm, whereas the random error of the lateral position is below 1.5 µm centered in front of the sensor. To increase the aperture and thus reduce the measurement uncertainty of the axial position component, the concept of using two shadow imaging sensors with overlapping measurement regions for the tool tip position measurement is proposed. However, the capability of a triangulation approach using shadow imaging sensors for 3D position measurement in the ISF machining volume is not clear, yet. To solve this issue, the question arises which uncertainty is achieved in which measuring volume when the measuring regions of two sensors overlap. Additionally, the limits of the measurement range that one sensor can cover and the different contributions to the measurement uncertainty budget, including the sensor calibration, must be explored.

### 1.3. Aim and Outline

The aim of the present article is to propose a triangulation approach based on shadow imaging sensors for measuring 3D tool deflection in incremental sheet forming. Hence, the measurement regions of two sensors overlap and the 3D tool tip position is measured using triangulation. On the one hand, the 3D measuring volume that two sensors are capable of covering is identified. On the other hand, the achievable measurement uncertainty of the three-dimensional tool tip position is assessed. The measurement uncertainty results from optical and geometric influences, which also affect the calibration. To reveal further optimization potential, the effects of these influences are investigated.

In the following, the 3D tool deflection measuring principle by means of a light-emitting diode as the point light source and two or more shadow imaging sensors is introduced in Section 2. Section 3 presents the experimental setup that is subsequently used to investigate the dynamic range of a two-sensor system. Studying the achievable measurement range and the measurement uncertainty, respective experimental results are shown and discussed in Section 4. Finally, Section 5 gives a conclusion and an outlook.

## 2. Principle of Measurement

To apply the shadow imaging principle for tool deflection measurement in ISF, a point light source is attached to the tool tip. For the determination of the light source position $l={\left(x_{L},y_{L},z_{L}\right)}^{T}$, two or more shadow imaging sensors are used, each of which consists of a mask and a camera chip. The light source casts a shadow through the mask on the camera chip. By evaluating the shadow position on the camera chip in the image, each sensor measures the direction to the light source. Note that a real light source is not punctual, but during the shadow position evaluation, the average shadow positions of mask features are obtained so that the resulting direction points to the center of the light source. Based on the shadow position evaluation, each sensor n=1,...,N provides possible light source positions ${l_{n}}^{\prime }={\left(x_{L,n}^{\prime },y_{L,n}^{\prime },z_{L,n}^{\prime }\right)}^{T}$ that are arranged in a line:

(1) $${l_{n}}^{\prime }=s_{n}+t\cdot r_{m,n}.$$

**Figure 1:**
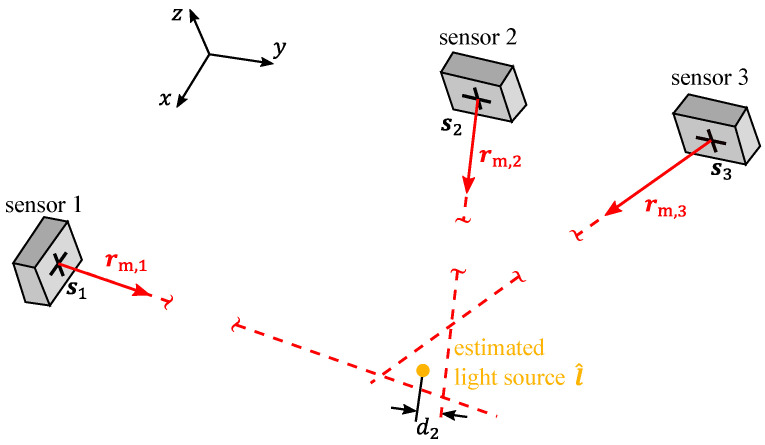
Principle of measuring the light source position using the combination of two or more (here exemplarily three) sensors. The best estimate of the light source position $\hat{l}$ is the point with the closest squared distance $d_{n}$ to the red dashed lines, each of which is given by one sensor and contains possible light source positions. The distance $d_{2}$ between sensor n=2 and the estimated light source position is exemplarily shown. Each sensor n=1,...,N provides one line that is determined by the sensor’s position $s_{n}$, marked by a black cross and the evaluated direction vector $r_{m,n}$ in the (x,y,z) machine coordinate system.

This line is defined by the sensor’s position $s_{n}={\left(x_{s,n},y_{s,n},z_{s,n}\right)}^{T}$, which is known from a calibration, and the measured direction vector $r_{m,n}={\left(r_{x,n},r_{y,n},r_{z,n}\right)}^{T}$ in the (x,y,z) machine coordinate system. The scalar parameter *t* leads to a certain point on the line. In practical 3D measurements, the lines measured with *N* sensors probably do not intersect at one point, which is shown in Figure 1 for a combination of three sensors. Note that the experimental investigations in this paper focus on the combination of two sensors per sub-region of the measurement range. For every number of sensors, the best estimate $\hat{l}$ of the sought light source position finally follows from the point with the closest squared distances $d_{n}$ to all lines, i.e., by calculating

(2) $$\min_{\hat{l}}\left(\sum_{n=1}^{N}{\left(d_{n}\left(\hat{l}\right)\right)}^{2}\right)$$

with

(3) $$d_{n}\left(\hat{l}\right)=\frac{|\left(\hat{l}-s_{n}\right)\times r_{m,n}|}{|r_{m,n}|}.$$

As a result, for measuring the 3D light source position, it is necessary to determine the sensor positions $s_{n}$ via calibration and to extract the direction vectors $r_{m,n}$ from two or more sensors to the light source.

For a detailed explanation of how the direction vectors are obtained, only one sensor is considered, and the index *n* specifying the sensor number is omitted in the following. The direction vector $r_{m}$ in machine coordinates is obtained via a coordinate transformation of the direction vector $r_{s}={\left(r_{\xi },r_{\eta },r_{\zeta }\right)}^{T}$ that is detected in the (ξ,η,ζ) sensor coordinate system. The coordinate transformation is a rotation by the angle γ around the *z*-axis, then by the angle β around the *y*-axis and lastly by the angle α around the *x*-axis:

(4) $$r_{m}=R_{\alpha }\cdot R_{\beta }\cdot R_{\gamma }\cdot r_{s},$$

i.e., the elementary rotation matrices $R_{\alpha }$, $R_{\beta }$ and $R_{\gamma }$, based on the respective rotation angles α, β and γ, which are obtained from the sensor calibration, are applied. A possible misalignment between the mask and the camera is neglected, as the tilt is minimized by grooves that place the mask and the mask rotation is corrected based on the camera image.

**Figure 2:**
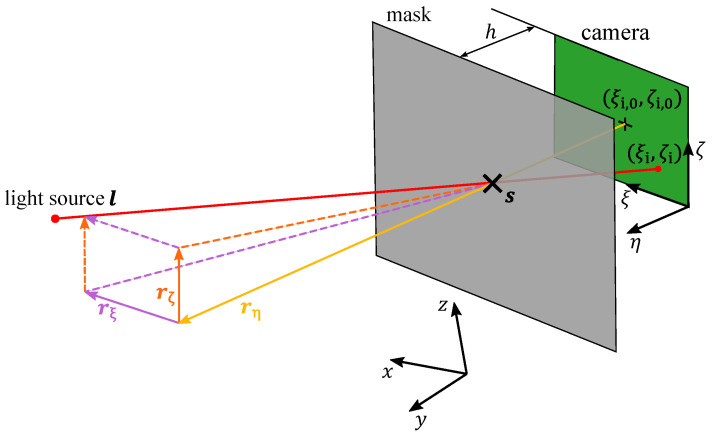
Principle of a single shadow imaging sensor. The light source at position l projects the shadow of a mask onto a camera chip. The shadow position $\left(\xi_{i},\zeta_{i}\right)$ is the position in the (ξ,ζ) image plane where the shadow of the mask center, i.e., the sensor position s highlighted by a black cross, appears. The direction vector $r_{s}$ in sensor coordinates, shown by its components $r_{\xi }$, $r_{\eta }$ and $r_{\zeta }$, points to the light source.

The direction vector in sensor coordinates

(5) $$r_{s}=\left[\begin{array}{c} \xi_{i,0}-\xi_{i}\\ h\\ \zeta_{i,0}-\zeta_{i}) \end{array}\right]$$

results from the shadow position $\left(\xi_{i},\zeta_{i}\right)$ detected in the camera image and calibrated intrinsic sensor parameters, namely the shadow position $\left(\xi_{i},0,\zeta_{i},0\right)$ when the light source is centered in front of the sensor and the distance *h* between the mask and the sensor. The relation between the shadow position $\left(\xi_{i},\zeta_{i}\right)$ and the direction vector $r_{s}={\left(r_{\xi },r_{\eta },r_{\zeta }\right)}^{T}$ in sensor coordinates including the sensor calibration parameters is visualized in Figure 2. As a result of Equations (4) and (5), each shadow imaging sensor finally provides the direction to the light source in machine coordinates. The position of the light source attached to the tool tip is then determined with the sensors’ output and the calibrated sensors’ positions by applying Equations (2) and (3).

## 3. Methods

### 3.1. Shadow Imaging Sensor

**Figure 3:**
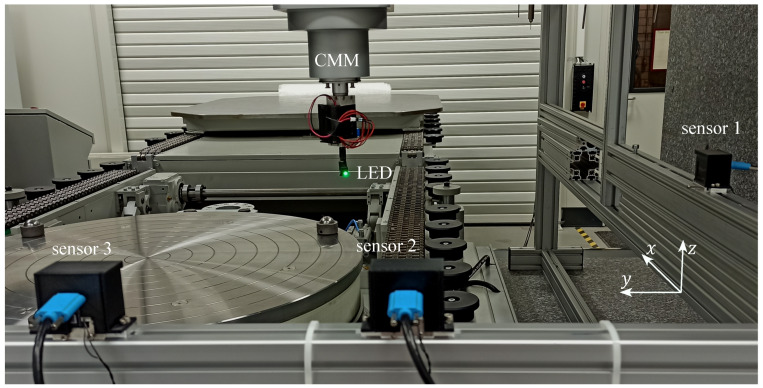
Experimental setup to investigate the 3D position measurement capability of a measurement system of several shadow imaging sensors in a measurement volume of 500 mm × 300 mm × 200 mm. The LED is positioned using a coordinate measuring machine (CMM), which also serves as reference system.

For the experimental investigation of the 3D position measurement capability, a minimal setup with a light source and three shadow imaging sensors is used, see Figure 3. The required measuring volume per sensor is investigated for sensor 1, and the measuring volume is divided in two sub-regions where sensor 2 or sensor 3, respectively, provides the second sensor for triangulation. The light source, whose position is to be measured, is a surface-mounted device LED type 0805 from the brand WINGER with a peak wavelength of 520 nm, a maximum luminous intensity of 1300 mcd and a beam angle of 140°. The LED is significantly smaller than, e.g., reflectors for laser trackers or targets for photogrammetry, which is, on the one hand, more susceptible to be covered by other objects in the process environment, but on the other hand can be attached closer to the tool tip, and the position measured is averaged over a smaller area. Each sensor consists of a 30 mm × 40 mm large mask with transparent and opaque parts, which is manufactured by laser exposure of a polyester film, and a DMM 37UX273-ML monochrome board camera from the company The Imaging Source. The camera has a resolution of 1440 px × 1080 px with a pixel size of 3.45 µm. The resolution is less than typically used for photgrammetry, which is affordable, because in shadow imaging, a higher content of the pixels contains information on the tool position. The distance *h* between mask and camera is 20 mm. With this sensor design, a lateral LED shift of 50 µm at a measuring distance of at least 300 mm leads to a shadow position shift, i.e., a motion blur, of less than 1 pixel.

#### 3.1.1. Mask

**Figure 4:**
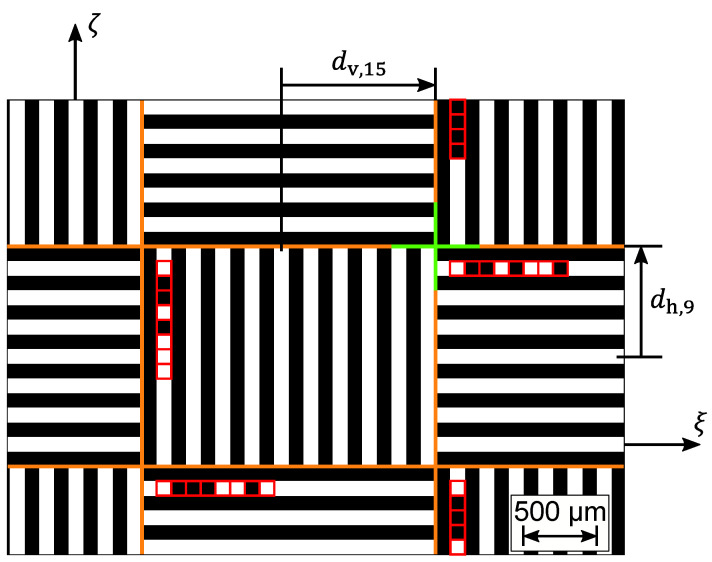
Section of the mask used in the shadow imaging sensors. The mask is the black and white structure, wherein black areas represent opaque contents and white areas transparent contents. The orange lines highlight the borders between the horizontal and vertical grids. The red squares visualize the bits used to build the binary index of each grid. The center of the entire mask is marked by the green cross. The axes ξ and ζ are projected from the sensor coordinate system to the mask plane. The distances $d_{v,a}$ and $d_{h,b}$ in the mask plane between the mask center and the stripes with the indexes *a* and *b*, respectively, are known. As an example, the distances $d_{v,a}$ and $d_{h,b}$ for the stripes *a* = 15 and *b* = 9 are visualized.

For measuring the absolute 3D position of the tool tip, i.e., the LED, a mask is required that contains features in horizontal and vertical direction and absolute features. A section of the used mask is shown in Figure 4. The mask contains alternately arranged grids with vertical and horizontal stripes. Vertical stripes enable one to determine the horizontal shadow position $\xi_{i}$ and horizontal stripes allow for the evaluation of the vertical shadow position $\zeta_{i}$, respectively. In contrast to circular markers or random patterns, grids allow for averaging in one direction over a large area and thus decrease time-consumption for image processing, which increases the potential for real-time measurement. In order to ensure that at least one full grid is always visible in the image while the LED is moved through the entire measurement volume, each grid has a size of 2.0 mm × 1.5 mm. Each stripe in a grid is 100 µm wide. The absolute feature is realized by 8-bit binary codes in each first transparent stripe of a grid. Eight adjacent squares are either transparent and provide a ‘0’ or opaque and provide a ‘1’ and so form an index of the grid. In the mask, each index is used twice, once for a vertical grid and once for a horizontal grid. The index defines where each grid is located with respect to the mask center. Therefore, the coded grid mask enables the determination of the absolute shadow position of the mask center in horizontal and vertical direction so that the absolute 3D LED position can be measured by two or more sensors.

#### 3.1.2. Image Processing

For the investigated shadow imaging sensors, cameras with a relatively low resolution, i.e., a low amount of data per image, are chosen, which offers the potential for real-time image processing and thus enables the active control of the forming process in future. To determine the position where the shadow of the mask center occurs in the image plane, the grids must be segmented first. In a second step, the stripes in each grid are localized, and then the index is read in the binary coded stripe. The position of the shadow of the mask center is then obtained by evaluating the location of the shadows of the stripes visible in the image, the location of these stripes in the mask with respect to the mask center and the magnification of the stripe spacing in the shadow image with respect to the stripe spacing in the mask.

**Figure 5:**
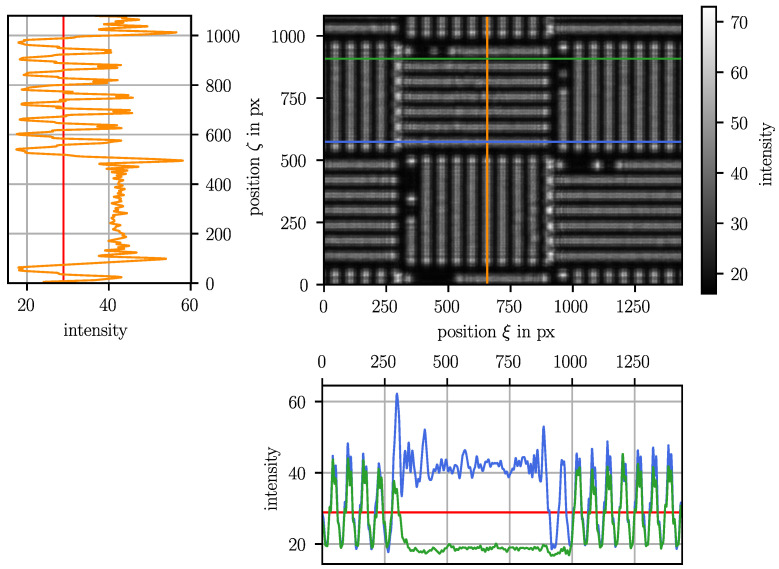
Camera image with intensity profiles used for grid segmentation. The lines in the image are the columns or rows where the filtered intensity profiles shown in the same color are taken. The red line in each intensity graph presents the threshold intensity. The intersections of the orange intensity profile and the threshold next to high-level-plateaus are horizontal borders. The left vertical border is located where the green intensity profile crosses the threshold on the left of the low-level-plateau and the right vertical border is located where the blue intensity profile crosses the threshold on the right of the high-level-plateau.

To separate the grids, a threshold method is applied that detects the horizontal and vertical borders. For visualization, an example image with evaluated intensity profiles is shown in Figure 5. Horizontal borders are located as the drop of the intensity after a bright vertical stripe. A vertical stripe is detected as a peak in the column-wise averaged intensity. A previously performed low-pass-filtering ensures robustness of the image processing against noise. Then, the horizontal borders are located in the row, where the filtered column intensity first passes through a threshold intensity after a plateau on a higher level. Here, the threshold intensity is the average intensity of the entire image and the intensity plateau indicates a stripe of a vertical grid. A respective intensity profile is given by the orange profile in Figure 5. Similarly, vertical borders are detected. A right border of a horizontal grid is where the filtered intensity passes through a threshold on the right side of the high-level-plateau, i.e., a horizontal bright stripe in the image, see the blue intensity profile in Figure 5. Accordingly, the left border of a horizontal grid is where the intensity passes through the threshold on the left side of a low-level-plateau, i.e., a dark horizontal stripe, as shown by the intensity profile in Figure 5.

In each grid, the stripes are localized separately by approximating a model function because preliminary investigations have shown that this method provides more accurate results than a phase evaluation based on a fast Fourier transform or a correlation [24]. Before the approximation, the image section is averaged in the direction of the stripes and a low-pass-filter is applied to smooth the interferences due to noise and diffraction. Then, the location of each stripe is determined in the (ξ,η,ζ) sensor coordinate system which is aligned to the plane of the camera chip. For this purpose, the intensity profile of a bright stripe in the region between adjacent intensity minima is approximated by the model function. For each vertical bright stripe *a*, the applied model function over the horizontal image coordinate ξ is

(6) $$I_{M,v}\left(\xi \right)=\left\{\begin{array}{cc} {I^{\prime }}_{M,v}\left(\xi \right) & \mathrm{for}\;{I^{\prime }}_{M,v}\left(\xi \right)<I_{\max,v,a}\\ I_{\max,v,a} & \mathrm{for}\;{I^{\prime }}_{M,v}\left(\xi \right)\geq I_{\max,v,a} \end{array}\right.$$

with

$${I^{\prime }}_{M,v}\left(\xi \right)=I_{0,v,a}+A_{v,a}\cdot e^{-{\left(\frac{|\xi -\mu_{v,a}|}{w_{v,a}}\right)}^{2}}$$

and for each horizontal bright stripe *b*, the model function over the vertical image coordinate ζ is

(7) $$I_{M,h}\left(\zeta \right)=\left\{\begin{array}{cc} {I^{\prime }}_{M,h}\left(\zeta \right) & \mathrm{for}\;{I^{\prime }}_{M,h}\left(\zeta \right)<I_{\max,h,b}\\ I_{\max,h,b} & \mathrm{for}\;{I^{\prime }}_{M,h}\left(\zeta \right)\geq I_{\max,h,b} \end{array}\right.$$

with

$${I^{\prime }}_{M,h}\left(\zeta \right)=I_{0,h,b}+A_{h,b}\cdot e^{-{\left(\frac{|\zeta -\mu_{h,b}|}{w_{h,b}}\right)}^{2}},$$

respectively. The model function is a limited Gaussian function with an offset $I_{0}$, an amplitude *A*, a width *w*, a peak position μ and an intensity limit $I_{\max}$. The index v refers to a vertical stripe and the index h to a horizontal stripe. For the approximation of the model function between adjacent minima, each pixel with its intensity provides one data point, to which the model function is fitted using a non-linear least squares approach. With the approximation, the parameters of the model function are determined. The resulting position $\mu_{v,a}$ serves as ξ-stripe location for a vertical stripe *a* and the determined peak position $\mu_{h,b}$ is the ζ-stripe location of a horizontal stripe *b*. Applying this approach, the stripe locations are obtained with subpixel resolution.

To calculate the absolute shadow position, the index of one grid in the image is needed. The locations of stripes in adjacent grids are used to determine the borders of each code bit. The intensity averaged in the quadratic range of each bit of the coded line is compared with an empirical threshold, which adapts to the image intensity. Mean bit intensities higher than the threshold are associated with a ’0’ and lower intensities provide a ’1’, and thus, the index is composed of the code bits.

The determined index enables to calculate the shadow position of the mask center. Indeed, using the index, each stripe in the image can be associated with a stripe in the mask whose absolute position with respect to the mask center is known. To transfer the mask stripe position with respect to the mask center to the image plane, the magnification

(8) $$k=\frac{l_{S}}{l_{M}}$$

of the stripe spacing $l_{S}$ in the shadow on the camera chip with respect to the stripe spacing $l_{M}$ in the mask is applied. Using the stripe position *d* in the mask, the magnification *k* and the location μ of the stripe shadow, each stripe donates an estimation for the mask center shadow position. As a result, the mask center shadow position is calculated by averaging the estimations. For the horizontal and vertical coordinates, this means that the horizontal mask center shadow position

(9) $$\xi_{i}=\frac{1}{s_{v,1}-s_{v,0}+1}\cdot \sum_{a=s_{v,0}}^{s_{v,1}}\left(\mu_{v,a}+d_{v,a}\cdot k\right)$$

and the vertical mask center shadow position

(10) $$\zeta_{i}=\frac{1}{s_{h,1}-s_{h,0}+1}\cdot \sum_{b=s_{h,0}}^{s_{h,1}}\left(\mu_{h,b}+d_{h,b}\cdot k\right)$$

are calculated from the stripe shadow positions $\mu_{v,a}$ of each vertical stripe *a* or $\mu_{h,b}$ of each horizontal stripe *b* visible in the image, the positions $d_{v,a}$ in the horizontal direction or $d_{h,b}$ in the vertical direction of each stripe in the mask, and the magnification *k*. Here, $s_{v,0}$ is the first and $s_{v,1}$ the last index of the vertical stripes in the image, and $s_{h,0}$ is the first and $s_{h,1}$ the last index of the horizontal stripes. This way, the absolute shadow position $\left(\xi_{i},\zeta_{i}\right)$ is evaluated for each image. The shadow position is then inserted into Equations (4) and (5) to obtain the direction vector $r_{m,n}$ pointing from the sensor to the LED, and the measured directions to the LED from several sensors finally provide the sought LED position according to Equations (2) and (3).

### 3.2. Experimental Setup with Three Sensors

In the experiments, sensor 1 is investigated in an axial measurement range of 500 mm beginning at a minimum measuring distance of 300 mm. The investigated lateral measurement range is 300 mm in horizontal direction and 200 mm in vertical direction, each centered in front of the sensor. Sensor 2 and sensor 3 are oriented perpendicular to sensor 1, and sensor 2 serves as second sensor for triangulation in the closer half of the axial measurement range of sensor 1, whereas sensor 3 covers the farther half. The investigated measurement range is located at a distance of 400 mm in front of sensor 2 and sensor 3. The perpendicular sensor arrangement is chosen because it is expected that a lower sum of the squares of the position component uncertainties is achieved if the angle between two measured direction vectors is close to 90° and if the angle of view from the sensor to the LED is close to 0° [22]. The LED is oriented at an angle of 45° towards the negative *x*- and *y*-axis so that the LED illuminates all sensors. For future applications in ISF, a clamping might conceal the tool and thus the LED. A solution for this is a tilted sensor setup, which is feasible due to the sensor’s three-dimensional position measurement capability.

During the experiment, the LED is moved step-wise by the coordinate measuring machine Leitz PMM-F 30.20.7, which simulates the forming tool in ISF and simultaneously serves as reference. At each position, ten images are recorded with an exposure time of 25 ms. Note that a flashing high-power LED can be used in future to meet the required measurement duration of 1 ms. In the first step, the LED is moved to defined positions to calibrate the sensor parameters. By recording ten images, the random error is reduced by averaging and thus the accuracy of the calibration is improved. In the second step, the LED is moved to a set of positions to investigate the measurement uncertainty. Here, ten images per position are recorded to study systematic and random errors.

**Figure 6:**
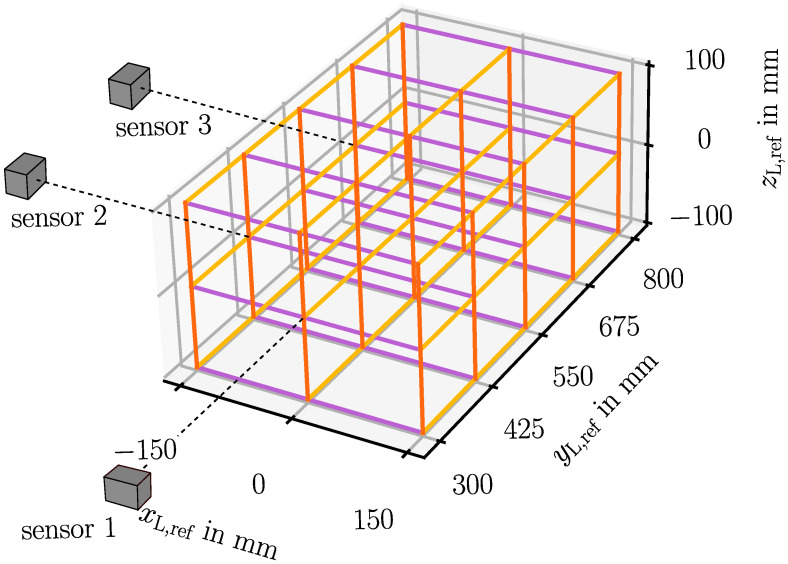
Paths on which the LED is moved in order to evaluate the 3D position measurement uncertainty. The lines are oriented parallel to the machine coordinate axes *x*, *y* and *z*.

In the uncertainty investigation, the LED is moved along the paths shown in Figure 6, which are arranged parallel to the global *x*-, *y*- and *z*-axis. Images are captured every 10 mm where the LED movement stops. Therefore, the uncertainty of the 3D position measurement by means of triangulation of two shadow imaging sensors can be evaluated in dependence of the LED location in a measurement volume of 500 mm × 300 mm × 200 mm, which is sufficient with respect to the application in a multi-sensor system in ISF.

### 3.3. Calibration

According to the sensing principle explained in Section 2, the LED position is calculated from the shadow positions of two or more sensors. For this purpose, the relation between the shadow position and the line of possible LED positions must be calibrated for each sensor. One calibration option is to record a full calibration map and the other option is to conduct a model-based calibration in which the geometrical quantities are determined. Since a calibration map requires the evaluation of shadow positions assigned to multiple LED positions that are arranged in a fine grid, this method is time-consuming, especially in a three-dimensional measuring volume. In addition, the interpolation between the LED positions might lead to deviations because of the non-linear relations and its dependence on unknown geometrical parameters. Instead, the model-based calibration is preferred here due to the lower number of LED positions in the calibration process.

For the model-based calibration, each sensor is calibrated separately, whereby a grid of LED positions is recorded. The positions are arranged in planes approximately parallel to the image plane of the sensor. The distance between adjacent positions in horizontal and vertical lateral direction is 20 mm, and the axial distance between the planes is 33.3 mm. For the future implementation of a calibration procedure in the ISF machine tool, a calibration target could be realized on which LEDs are arranged in a two-dimensional grid. The target can be moved in defined steps whereby the LEDs blink in sequence. In contrast to the calibration map, the distance between the adjacent points is larger, which significantly reduces the number of LED positions required. The axial and lateral range of the calibration volume is adjusted to the intended measurement range of the sensor. In the first step, the sensor position $s_{n}$ is evaluated as the intersection of lines fitted in LED positions that provide the same shadow positions. For this purpose, the LED position of each plane belonging to a certain shadow position is obtained via a regression in the calibration plane. In the second step, the geometrical model according to Equations (4) and (5) is fitted to the direction vectors pointing from the extracted sensor position to the defined LED positions. As a result, the remaining sensor parameters are obtained, i.e., the distance *h* between mask and sensor, the shadow position $\left(\xi_{i,0},\zeta_{i,0}\right)$ that belongs to LED positions centered in front of the sensor and the sensor orientation (α,β,γ) in the machine coordinate system.

## 4. Results and Discussion

### 4.1. Measurement Range

The point grid of LED positions captured during the calibration of sensor 1 is also used to evaluate the measurement range of each shadow imaging sensor. To evaluate the limits of the measurement range, two criteria are considered, the contrast-to-noise ratio (CNR) and the detectability of all stripes in the image using the algorithm described in Section 3.1.2. The CNR is the main limiting factor of the measurement range, which here is defined as

(11) $$\mathrm{CNR}=\frac{{\bar{I}}_{95}-{\bar{I}}_{5}}{\bar{s}}$$

to characterize the quality of the images. The CNR is evaluated based on all ten images captured at the same LED position. Therefore, the intensity is averaged over all images and the contrast is measured by the difference between the 95% percentile ${\bar{I}}_{95}$ and the 5% percentile ${\bar{I}}_{5}$ of the average intensity, and the noise $\bar{s}$ is the averaged standard deviation per pixel. The percentile ensures that rare pixels with very high or low intensities are excluded, so that the contrast represents the main characteristic of each image. However, at large angles of view, the stripes might not be detected despite a high CNR, because the stripe intensity profile changes due to diffraction effects.

**Figure 7:**
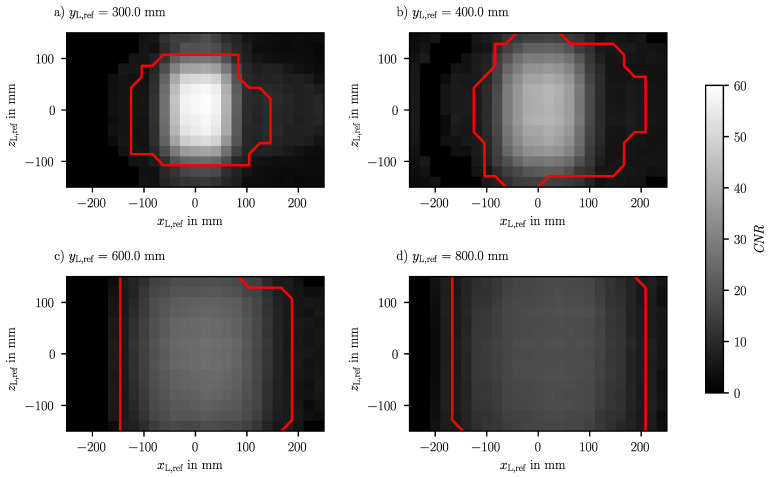
Contrast-to-noise ratios (CNRs) calculated in the images during the calibration of sensor 1 depending on the LED position. The red lines show the boundaries within which valid shadow positions are evaluated from the images. The graphs contain the results for the planes at (**a**) $y_{L,\mathrm{ref}}=$ 300 mm, (**b**) $y_{L,\mathrm{ref}}=$ 400 mm, (**c**) $y_{L,\mathrm{ref}}=$ 600 mm and (**d**) $y_{L,\mathrm{ref}}=$ 800 mm.

The resulting CNR and the identified boundaries of the lateral measurement range are given in Figure 7 for different axial distances to sensor 1 from 300 mm to 800 mm. The largest CNR occurs centered in front of the sensor at the closest axial distance and decreases sharply in the lateral direction. Here, the valid lateral measurement range is the smallest with 280 mm in the horizontal direction and 220 mm in the vertical direction, but the outer corners are outside the measurement range. This means that the measurement range of 200 mm in the lateral direction is fully covered. Although the CNR decreases with an increasing axial distance, the lateral measurement range increases because the CNR drops less sharply in the lateral direction. At $y_{L,\mathrm{ref}}=$ 400 mm, the outer corners are still not covered, but at $y_{L,\mathrm{ref}}\geq$ 600 mm, the measurement range is mainly shaped rectangularly and a lateral extent of 300 mm is achieved. Additionally, the maximum CNR is not centered in front of the sensor but is slightly shifted in the positive *x*-direction as the achieved measurement range which corresponds to an according shift in the measurement range.

In summary, the measurement range of each sensor is primary limited by the CNR of the images. To further increase the CNR, a brighter LED or a longer exposure could be used. Note that ambient light, which is relevant in ISF applications, indeed decreases the CNR, but this effect can be reduced by applying a bandpass filter. The angle of incidence is the dominant affect on the CNR, which means that a larger lateral measurement range is covered at larger axial distances. Another significant influence is the axial distance, but even at the largest axial distance, the CNR is sufficient for the evaluation of the shadow position. As a result, the aimed lateral measurement range of 200 mm is reached and an enlargement of the measurement range in the axial direction is possible. Finally, each point in the machining volume must be covered by at least two sensors to measure the tool tip position in the ISF. This is evidenced by the proven axial measurement range of at least 500 mm.

### 4.2. Random Error

The paths shown in Figure 6 are used to assess the random and systematic measurement error of the three-dimensional LED position. The random measurement error is given by the standard deviation of the measured LED positions and is subsequently considered for each position component separately.

**Figure 8:**
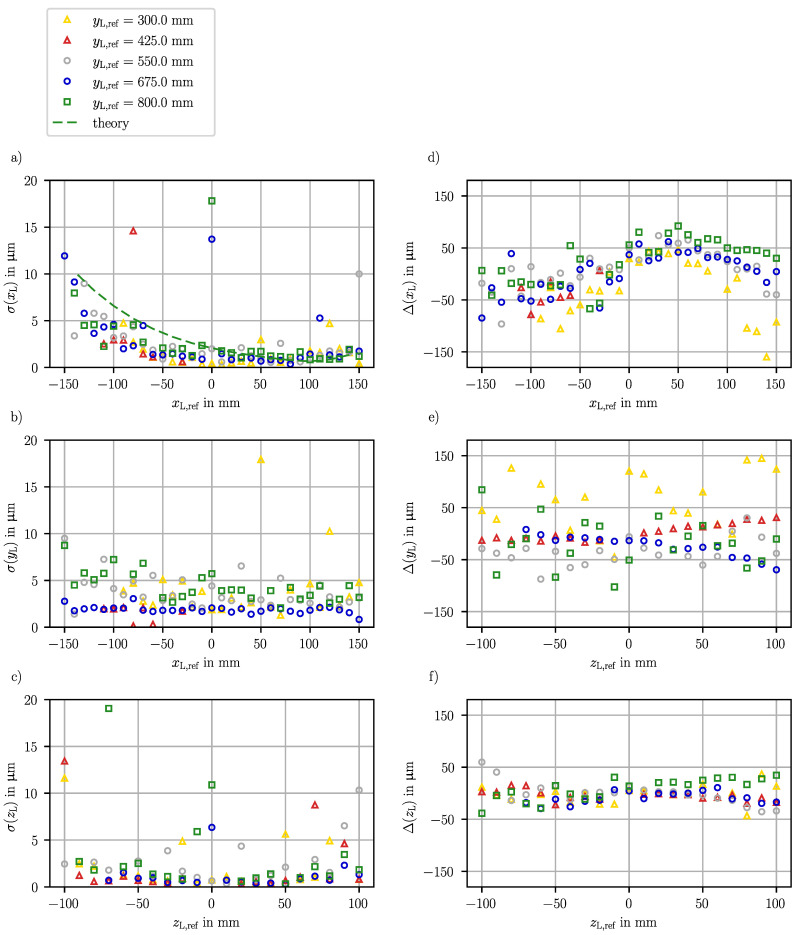
Random errors σ and systematic errors Δ of the measured LED position components $\left(x_{L},y_{L},z_{L}\right)$ evaluated in the test data set for various $y_{L,\mathrm{ref}}$: (**a**) random error $\sigma (x_{L})$ over $x_{L,\mathrm{ref}}$ coordinates at $z_{L,\mathrm{ref}}=$ 0 mm, including the theoretical course calculated for $y_{L,\mathrm{ref}}=$ 800 mm based on an uncertainty propagation; (**b**) random error $\sigma (y_{L})$ over $x_{L,\mathrm{ref}}$ coordinates at $z_{L,\mathrm{ref}}=$ 0 mm; (**c**) random error $\sigma (z_{L})$ over $z_{L,\mathrm{ref}}$ coordinates at $x_{L,\mathrm{ref}}=$ 0 mm. In the random error, outliers occur due to the random detection or non-detection of stripes at the image edges. (**d**) Systematic error $\Delta (x_{L})$ over $x_{L,\mathrm{ref}}$ coordinates at $z_{L,\mathrm{ref}}=$ 0 mm, (**e**) systematic error $\Delta (y_{L})$ over $z_{L,\mathrm{ref}}$ coordinates at $x_{L,\mathrm{ref}}=$ 0 mm, (**f**) systematic error $\Delta (z_{L})$ over $z_{L,\mathrm{ref}}$ coordinates at $x_{L,\mathrm{ref}}=$ 0 mm.

The position component $x_{L}$ is directed horizontally lateral to sensor 1. Its random error $\sigma (x_{L})$ in dependence of the position component $x_{L,\mathrm{ref}}$ horizontally lateral to sensor 1 is shown in Figure 8a, wherein the included paths are located at $z_{L,\mathrm{ref}}=$ 0 mm, i.e., vertically centered in front of sensor 1 and at various $y_{L,\mathrm{ref}}$ coordinates. The missing random errors at $y_{L,\mathrm{ref}}=$ 300 µm at small $x_{L,\mathrm{ref}}$ result from the limitation of the measurement range. Additonally, at several positions on the path at $y_{L,\mathrm{ref}}=$ 425 mm, invalid indexes were evaluated which lead to invalid shadow positions so that the LED position calculation is not possible. Larger coded bits would increase the robustness of the algorithm and prevent invalid results in future. Nevertheless, most of the LED positions provide valid results that contribute to the error evaluation. Among the evaluated random errors $\sigma (x_{L})$ on paths along the *x*-axis at $z_{L,\mathrm{ref}}$ 0 mm, 80% are below 4 µm. However, a significant tendential increase in the random error $\sigma (x_{L})$ towards small $x_{L,\mathrm{ref}}$ coordinates is prominent. In addition, a few randomly occuring higher random errors are present.

To reveal the causes for the principle course of the random error, the uncertainty budget is discussed in detail in Appendix A. The results of the theoretical model for the position component $x_{L}$ at $y_{L,\mathrm{ref}}=$ 800 mm are also included in Figure 8a. The shadow imaging sensors mainly determine the respective lateral position component, and according to Equation (A2), the uncertainty of that position component depends on the uncertainty of the evaluated shadow position and the axial distance to the LED. Thereby, the shadow position uncertainty is dominated by the angle of view which linearly affects the propagation of the magnification uncertainty (see Equation (A4)). The contribution of the shadow position uncertainty to the lateral position uncertainty increases with the axial distance. However, the effect of the axial distance is smaller than the effect of the angle of view.

The experimentally evaluated random error $\sigma (x_{L})$ in Figure 8a validates that the angle-dependent increase in the error due to the propagation of the magnification uncertainty is the dominant effect. The slight increase in the random error $\sigma (x_{L})$ with an increasing $y_{L,\mathrm{ref}}$ at the inner lateral positions proves the dependence on the axial distance. Since the axial distance dependency is a minor effect, it will not be investigated further. Additionally, the magnitude of the experimentally evaluated error corresponds to the theoretically propagated uncertainty. The remaining deviations between the propagated uncertainty and the evaluated random error result from averaging the uncertainty of the stripe location, the assumptions and simplifications considering the calibration parameters and the deviations of sensors 2 and 3 affecting the axial position component. An investigation of outlying high random errors revealed that the reason for the outliers are stripes on the outside of an image that are detected in some but not all images captured at the same LED position and strongly affect the calculation of the magnification.

The same angle-dependent increase is expected for all position components. The random error $\sigma (y_{L})$ along the *x*-axis for various $y_{L,\mathrm{ref}}$ coordinates on the vertically centered plane at $z_{L,\mathrm{ref}}$ 0 mm is shown in Figure 8b. At the positions laterally outside from the perspective of sensors 2 and 3, at $y_{L,\mathrm{ref}}=$ 300 mm, $y_{L,\mathrm{ref}}=$ 550 mm and $y_{L,\mathrm{ref}}=$ 800 mm, the random error $\sigma (y_{L})$ is higher than centered in front of the sensors at $y_{L,\mathrm{ref}}=$ 425 mm and $y_{L,\mathrm{ref}}=$ 675 mm. Moreover, the random error $\sigma (y_{L})$ decreases at the outer lateral positions with an increasing axial distance, which highlights the dominant angle-dependent increase in the random error. However, the random error $\sigma (y_{L})$ is on a higher level than the random error $\sigma (x_{L})$, and despite additional outliers, 80% of the random errors $\sigma (y_{L})$ are below 5 µm.

Also, the random error $\sigma (z_{L})$ of the position component $z_{L}$ provided in Figure 8c shows a slight angle-dependent increase. Furthermore, the random error $\sigma (z_{L})$ is smaller than the random error $\sigma (x_{L})$, which is caused by the smaller measurement range, by the more centered minimum of the error and by the fact that all three sensors are significantly sensitive to the position component $z_{L}$, because the position component $z_{L}$ is vertically lateral to all sensors.

In summary, the dominant influence on the random error is the angle of view to the laterally measuring sensor because the contribution of the uncertainty of the magnification *k* increases with the angle. Nevertheless, the random error is lower than 5 µm at most of the tested reference LED positions $\left(x_{L,\mathrm{ref}},y_{L,\mathrm{ref}},z_{L,\mathrm{ref}}\right)$, which is one order of magnitude better than the required position measurement uncertainty of 50 µm. Therefore, the achieved random error proves the potential of shadow imaging sensors for application in a multi-sensor system for measuring the three-dimensional tool tip position in ISF.

### 4.3. Systematic Error

The systematic position measurement error is also evaluated for the $\left(x_{L},y_{L},z_{L}\right)$ position components separately and is presented in Figure 8. The systematic error $\Delta (x_{L})$ of the position component $x_{L}$ along the *x*-axis, i.e., lateral to sensor 1, shown in Figure 8d for various axial distances $y_{L,\mathrm{ref}}$ and at $z_{L,\mathrm{ref}}$ = 0 mm, ranges from −160 µm to 92 µm, and its course is similar to an inverted parabola. The maximum is shifted from the middle in front of sensor 1 to higher $x_{L,\mathrm{ref}}$ values, which corresponds to smaller horizontal shadow position components $\xi_{i}$. Furthermore, it stands out that the range between the minimum and maximum systematic error is higher the closer the LED is to sensor 1. A probable reason is that the systematic error depends on the angle to the LED, which is larger at shorter distances for constant lateral positions. In addition to the tendential course, the systematic error $\Delta (x_{L})$ scatters at small lateral positions $x_{L,\mathrm{ref}}$.

For the position component $y_{L}$, the systematic error $\Delta (y_{L})$ is shown over $z_{L,\mathrm{ref}}$ coordinates in Figure 8e for various $y_{L,\mathrm{ref}}$, all in the same axial distance to sensors 2 and 3 at $x_{L,\mathrm{ref}}=$ 0 mm. On these paths, the minimal systematic error $\Delta (y_{L})$ is −103 µm and the maximal is 145 µm. A strong scatter of the systematic error $\Delta (y_{L})$ occurs on the outer paths at $y_{L,\mathrm{ref}}=$ 300 mm and $y_{L,\mathrm{ref}}=$ 800 mm with a standard deviation of 54 µm or 46 µm, respectively. Centered in front of sensor 2 at $y_{L,\mathrm{ref}}=$ 425 mm and in front of sensor 3 at $y_{L,\mathrm{ref}}=$ 675 mm, the systematic error $\Delta (y_{L})$ barely scatters but, due to a slope in the systematic error over the $z_{L,\mathrm{ref}}$ position, the standard deviation of the systematic error $\Delta (y_{L})$ is between 15 µm and 20 µm. The tendential slope depending on the $z_{L,\mathrm{ref}}$ coordinate shows a cross-sensitivity between the position components vertically and horizontally lateral to the sensors.

The systematic error $\Delta (z_{L})$ of the $z_{L}$ component along the *z*-axis given in Figure 8f for various $y_{L,\mathrm{ref}}$ coordinates centered in front of sensor 1 at $x_{L,\mathrm{ref}}=$ 0 mm is, in total, more constant than the systematic error of the other position components and ranges from −43 µm to 60 µm.

In all position components, systematic errors occur. It is assumed that the model-based calibration does not cover all main influences on the position component. For example, the orientation between the mask and camera chip is not considered, yet. In addition, cross-sensitivities are justified by the orientation angles of the sensor alignment in the machine coordinate system and also the neglected orientation between the mask and camera chip. The tendential course, which is the largest contribution to the systematic error, can be compensated by the extension of the geometrical model or the application of empirically obtained polynomial correction functions. The correction can reduce the standard deviation of the systematic error below 10 µm on paths centered in front of the respective sensor in the respective axis. However, this correction does not reduce the detected scatter which results from the angle of view-dependent propagation of variations in the evaluated magnification *k* according to Equation (A4). Probable reasons for the scatter in the magnification *k* are manufacturing deviations in the mask, which can either be calibrated by an individual characterization or reduced through a more precise manufacturing process.

In summary, the evaluated systematic errors in the range between −150 µm and 150 µm prove that valid three-dimensional LED positions are measured by combining two perpendicular shadow imaging sensors. By compensating the parabolic course of the systematic error, the aimed measurement uncertainty of 50 µm is achieved in most of the measuring range. However, the angle of view strongly affects the propagation of magnification deviations and therefore limits the lateral measurement range in which a sufficient measurement uncertainty is reached. Although the experimental results show a larger systematic error than other optical measurement approaches, like photogrammetry, laser interferometry and laser triangulation, shadow imaging sensors benefit from being able to measure the position close to the tool tip from a single shot and do not require tracking the region of interest. Concurrently, the random error proves the great potential of shadow imaging sensors.

## 5. Conclusions and Outlook

In order to measure the three-dimensional tool tip position in ISF in a measuring volume of 2.0 m × 1.0 m × 0.2 m with a measurement uncertainty below 50 µm, a multi-sensor system is proposed. The multi-sensor system consists of shadow imaging sensors, each of which provides the direction vector to an LED attached to the tool tip, and the LED position is obtained by combining the sensor data. Therefore, the measuring volume is split in sub-regions where each is covered by at least two sensors. A minimal configuration of three shadow imaging sensors is experimentally investigated to reveal the system’s three-dimensional position measuring capability for a sub-region of 300 mm × 500 mm × 200 mm.

The conducted experiments show that the combination of two perpendicular shadow imaging sensors is capable of measuring the three-dimensional tool tip position. Hence, for one sensor, an axial measurement range of at least 500 mm is proven, whereas the lateral measurement range is about 300 mm but depends on the angle of view and thus increases with the axial distance. The measurement uncertainty achieved by combining two sensors is dominated by the systematic error which can be compensated. However, the main contribution to the systematic and the random error is the magnification evaluated in the images, which propagates to higher position uncertainties the larger the angle of view is. As a result, the angle of view limits the achievable measurement uncertainty, but with a compensation of the tendential course of the systematic error, the measurement uncertainty is sufficient for tool tip position measurement in ISF.

The presented work revealed the limits of the lateral measurement of the sensors but not yet the limits of the axial measurement range, which will be subject of future work. A further study will include the extension of the geometrical model to cover previously neglected quantities affecting the systematic error. Additionally, the potential to reduce the measurement uncertainty by integrating additional sensors per sub-region will be explored in future. After further developments and characterizations regarding the sensor in laboratory environments, the next step will be the transfer of the measurement system to the manufacturing environment. Under manufacturing conditions, it is substantial to validate an adapted calibration procedure and to overcome specific challenges such as machine vibrations or thermal fluctuations.

## Data Availability

Data will be made available on request.

## Abbreviations

The following abbreviations are used in this manuscript:

CMM	coordinate measuring machine;
CNR	contrast-to-noise ratio;
DIC	digital image correlation;
ISF	incremental sheet forming.

## Appendix A. Uncertainty Propagation for Lateral Position Components

In order to explain the behavior of the random error and to reveal the sources of the random error, the uncertainty budget is investigated in detail whereby the geometric model is simplified. Consequently, to evaluate the sources of the random error $\sigma (x_{L})$, the calculation of the direction vector of sensor 1, which is sensitive to $x_{L}$, is focused on, while the axial position component $y_{L}$, predominantly provided by sensors 2 and 3, is assumed to be exactly known. Additionally, the position of sensor 1 is approximated to $x_{s,1}=$ 0 mm, $y_{s,1}=$ 0 mm and $z_{s,1}=$ 0 mm and its orientation angles α, β and γ in the machine coordinate system are also approximately zero. Therefore, from Equations (1), (4) and (5), it follows that the position component

(A1) $$x_{L}\approx y_{L}\cdot \frac{\xi_{i,0}-\xi_{i}}{h}$$

is calculated from the distance *h* between the camera and mask, the center shadow position $\xi_{i},0$, which are both constant geometrical parameters, the position component $y_{L}$, and the evaluated shadow position component $\xi_{i}$, which is the main source of the random error $\sigma (x_{L})$.

To obtain the simplified model in Equation (A1), the known position component $y_{L}$ and the approximated position of sensor 1 are inserted in Equation (1) first. Consequently, the position component $x_{L}\approx \frac{y_{L}}{r_{y,1}}\cdot r_{x,1}$ is determined using the axial position $y_{L}$ and the *x*-component of the direction vector $r_{m,1}$ in machine coordinates measured by sensor 1. In the following, the index specifying the sensor will be omitted because only sensor 1 is considered. Resulting from the approximated angles α, β and γ, Equation (4) becomes $r_{m}\approx r_{s}$ and it follows with Equation (5) that $r_{x}\approx r_{\xi }=\frac{\xi_{i,0}-\xi_{i}}{h}$ and $r_{y}\approx r_{\eta }=1$ so that the lateral position is expressed depending on the evaluated shadow position, the axial position component and fixed geometrical parameters (see Equation (A1)).

The sought uncertainty of the lateral LED position

(A2) $$u\left(x_{L}\right)\approx \frac{y_{L}}{h}\cdot u\left(\xi_{i}\right)$$

caused by sensor 1 is the shadow position uncertainty $u(\xi_{i})$ scaled by the relation between the known axial position $y_{L}=y_{L,\mathrm{ref}}$ and the distance *h* between the mask and camera chip which results from the uncertainty propagation applied to Equation (A1). The shadow position component $\xi_{i}$, whose uncertainty affects the lateral position uncertainty $u(x_{L})$, is determined from the vertical stripes in the respective image according to Equation (9), where the shadow position is calculated for each stripe separately and then averaged. Due to the linear relations, the calculation can be expressed as

(A3) $$\xi_{i}=\bar{\mu_{v}}+\bar{d_{v}}\cdot k$$

based on the evaluated average stripe location $\bar{\mu_{v}}$ in the image, the average stripe position $\bar{d_{v}}$ in the mask, which is a constant geometrical parameter, and the evaluated magnification *k*. The uncertainty of the shadow position

(A4) $$u\left(\xi_{i}\right)=\sqrt{{\left(\bar{d_{v}}\cdot u\left(k\right)\right)}^{2}+u^{2}\left(\bar{\mu_{v}}\right)}$$

is composed of the contribution of the magnification uncertainty u(k) and the average stripe location uncertainty $u(\bar{\mu_{v}})$. Note that the covariances of the leftmost and the rightmost stripe, which contribute to the average stripe location and the magnification, are equal but inverted and thus neglected. Therein, the magnification

(A5) $$k=\frac{1}{2}\cdot \left(\frac{\mu_{v,s_{v,1}}-\mu_{v,s_{v,0}}}{d_{v,s_{v,1}}-d_{v,s_{v,0}}}+\frac{\mu_{h,s_{h},1}-\mu_{h,s_{h,0}}}{d_{h,s_{h,1}}-d_{h,s_{h,0}}}\right)$$

is practically calculated in the vertical and horizontal direction as magnification of the spacing between the outermost stripes $s_{v,0}$ and $s_{v,1}$ or $s_{h,0}$ and $s_{h,1}$, respectively, detectable in the image, whereby $\mu_{v}$ and $\mu_{h}$ are the locations of the stripes in the image and $d_{v}$ and $d_{h}$ are the positions of the respective stripes in the mask. The respective stripe locations are evaluated in the images and thus contribute to the random error. So, according to an analytic uncertainty propagation of the uncertainty of each stripe location $u\left(\mu_{v,s_{v,0}}\right)\approx u\left(\mu_{v,s_{v,1}}\right)\approx u\left(\mu_{h,s_{h,0}}\right)\approx u\left(\mu_{h,s_{h,1}}\right)\approx u\left(\mu \right)$, the uncertainty of the magnification

(A6) $$u\left(k\right)\approx u\left(\mu \right)\cdot \sqrt{2}\cdot \frac{1}{2}\cdot \sqrt{{\left(\frac{1}{d_{v,s_{v,1}}-d_{v,s_{v,0}}}\right)}^{2}+{\left(\frac{1}{d_{h,s_{h,1}}-d_{h,s_{h,0}}}\right)}^{2}}$$

is calculated from the distance $d_{v,s_{v,1}}-d_{v,s_{v,0}}$ in the mask between the vertical stripes that are the farthest from the outside of the image, the distance $d_{h,s_{h,1}}-d_{h,s_{h,0}}$ in the mask between the respective horizontal stripes and the stripe location uncertainty u(μ). The uncertainty of the average stripe location

(A7) $$u\left(\bar{\mu_{v}}\right)\approx \frac{1}{\sqrt{s_{v,1}-s_{v,0}+1}}\cdot u\left(\mu \right),$$

contributing to the shadow position uncertainty $u(\xi_{i})$, is affected by the number of vertical stripes $s_{v,1}-s_{v,0}+1$ detected in the image. So, the evaluated stripe locations in the image contribute to the uncertainty of the magnification *k* and to the uncertainty of the average stripe location $\bar{\mu_{v}}$ in the image, and therefore affect the uncertainty of the shadow position component $\xi_{i}$, which finally propagates to the LED position component $x_{L}$.

The theoretical uncertainty propagation to the LED position component $x_{L}$ is calculated for the exemplary path along the *x*-axis at $y_{L,\mathrm{ref}}=$ 800 mm. The stripe location uncertainty u(μ) is obtained by calculating the standard deviation of the each stripe’s location evaluated in the images captured by sensor 1 during the calibration, averaged per LED position and then approximated with a second degree polynomial. The evaluated uncertainty u(μ) varies in correspondence to the CNR, where a higher CNR is accompanied by a lower uncertainty of the stripe location. This means that the stripe location uncertainty u(μ) increases primarily with an increasing angle of view and secondarily with an increasing axial distance to the sensor.

To calculate the uncertainty of the magnification, approximate outermost stripe distances in the mask of $d_{v,s_{v,1}}-d_{v,s_{v,0}}=$ 4400 µm and $d_{h,s_{h,1}}-d_{h,s_{h,0}}=$ 3200 µm are applied. For calculating the uncertainty $u(\bar{\mu_{v}})$ of the average stripe location, a number of vertical stripes of $s_{v,1}-s_{v,0}+1=$ 25 are estimated. The resulting uncertainty u(k) of the magnification and the resulting uncertainty $u(\bar{\mu_{v}})$ of the average stripe location depend linearly on the stripe location uncertainty, whereby the contribution of the magnification uncertainty u(k) increases proportionally with the average stripe position $\bar{d_{v}}$ in the mask and thus with the angle of view. Since the average stripe position $\bar{d_{v}}$ in the mask is up to 6800 µm at $y_{L,\mathrm{ref}}=$ 800 mm, the uncertainty u(k) of the magnification contributes dominantly to the uncertainty $u(\xi_{i})$ of the shadow position. Note that the angle of view reaches even larger values for closer axial distances, which amplifies the propagation of the magnification uncertainty u(k). The minimum of the shadow position uncertainty $u(\xi_{i})$ corresponds to the respective minimum of the absolute average stripe position $\bar{d_{v}}$ in the mask and is shifted from the center of the camera chip to higher $x_{L,\mathrm{ref}}$ coordinates, which depends on the location of the mask with respect to the sensor. Then, the uncertainty of the lateral LED position is scaled by the axial position $y_{L}=y_{L,\mathrm{ref}}$ and the approximate distance h= 20 mm between the mask and camera chip. So, the principle course of the position uncertainty $u(x_{L})$, included in Figure 8a for $y_{L,\mathrm{ref}}=$ 800 mm, corresponds to the course of the shadow position uncertainty $u(\xi_{i})$. Due to the impact of the axial position coordinate $y_{L,\mathrm{ref}}$, the minimum lateral LED position uncertainty $u(x_{L})$ is larger at farther axial distances, but the angle-dependent increase dominates the uncertainty $u(x_{L})$.
